# Analysis of the Rotation Bending Test Method and Characterization of Unidirectional Carbon Fiber-Reinforced Polycarbonate Tapes at Processing Temperatures

**DOI:** 10.3390/polym16030425

**Published:** 2024-02-02

**Authors:** Daniel Laresser, Matei-Constantin Miron, Milan Kracalik, Felix Baudach, Zoltán Major

**Affiliations:** 1 Competence Center CHASE GmbH, Hafenstraße 47-51, 4020 Linz, Austria; matei.miron@chasecenter.at; 2 Institute of Polymer Product Engineering, Johannes Kepler University Linz, Altenberger Str. 69, 4040 Linz, Austria; zoltan.major@jku.at; 3 Institute of Polymer Science, Johannes Kepler University Linz, Altenberger Str. 69, 4040 Linz, Austria; milan.kracalik@jku.at; 4 Covestro Deutschland AG, B207, R428, 51365 Leverkusen, Germany; felix.baudach@covestro.com

**Keywords:** bending, unidirectional tape, high temperature, thermoplastic composite, polycarbonate

## Abstract

Bending is one of the dominant material deformation mechanisms that occurs during the forming process of unidirectional (UD) thermoplastic tapes. Experimental characterization of the bending behavior at processing temperatures is crucial to obtaining close-to-reality data sets for process analysis or material modeling for process simulation. The main purpose of this study is to characterize to a high degree of accuracy the temperature-dependent bending behavior of single and multi-ply specimens of carbon fiber-reinforced polycarbonate (PC/CF) UD tapes at processing temperatures, which implies a molten state of the thermoplastic matrix. The application of the rotation bending test using a customized fixture may come with systematic deviations in the measured moment that result from a pivot offset or an effective clearance that is unknown under realistic test conditions. The present research analyzes these effects with analytical methods, experimental investigations, and simulations using a finite element model. In this context, a compensation method for the toe-in effect is evaluated. With this approach, we were able to obtain reliable data and characterize the bending resistance within the desired processing window. The data reveal a major drop in bending resistance between 200 °C and 250 °C and a less significant decrease between 250 °C and 300 °C. Analysis of the thickness-normalized bending resistances indicates a non-linear relationship between specimen thickness and measured moment but an increasing shear-dominated characteristic at higher temperatures.

## 1. Introduction

Continuous fiber-reinforced thermoplastics (CFRTPs) are becoming increasingly prominent in the automated production of lightweight components due to their potential for rapid processing and circular material economy. In production, flat preforms built up from individual UD tapes or fabrics can be formed into a geometry under heat and pressure within seconds. Overmolding the composite base structure in an injection molding process enables its functionalization with geometrically complex features [1,2,3,4,5,6].

In today’s CFRTP component development, modeling and simulation of the forming process provides a powerful approach for defect prediction and verification, as well as optimization of manufacturability. To ensure accurate simulation results, material-specific input data are a basic prerequisite in addition to a suitable material modeling approach [7,8,9]. These material data must be determined under processing conditions. Depending on the type of thermoplastic matrix material, testing temperatures up to 400 °C are required [10]. At these temperatures, the material consists of continuous, stiff fibers embedded in a molten polymer matrix. Under these testing conditions, conventional testing methods, as known from the standard mechanical testing of composites [11], are no longer applicable due to the increased demands on temperature management, instrumentation, and specimen handling or clamping. However, the emergence of new CFRTP grades incentivizes the search for efficient and reliable testing methods for high-temperature material characterization.

During the forming process of composite laminates, several deformation mechanisms take place simultaneously. Bending, in-plane shear, and slippage (ply/ply interactions and tool/ply interactions) mechanisms can be classified as the three primary modes of deformation in UD composites that determine the forming result [9,12,13]. Moreover, simulation studies have shown that the temperature dependence of the composite material influences the geometric accuracy of the final component and that the bending rigidity plays a significant role in the formation of wrinkles [14,15,16].

Several researchers have already addressed different methods to characterize the thermo-coupled bending behavior of CFRTP. In general, the current test configurations can be categorized into approaches based on the Cantilever test, the Kawabata test, and the Vee-bending test [17]. Reviews of the characterization approaches used by several researchers to obtain experimental bending material data for molten thermoplastic composites are provided in the literature [17,18]. Furthermore, Fernandez et al. presented in their work the so-called column bending test (CBT), which is applicable for large-deformation bending testing of thin-ply coupons [19]. However, studies under high-temperature conditions are not yet available for the CBT approach. It has to be noted that obtaining bending material data at process-relevant levels of curvature, deformation rate, and temperature, as well as dealing with low specimen integrity at high temperatures, is a challenging task and is not achievable with every test approach presented in the literature.

The Kawabata-inspired bending test, proposed by Sachs et al. [20,21] (referred to as the rotation bending test in this study), is gaining increasing acceptance in the scientific community. It is based on a custom-built fixture applied to a commercial rheometer. The rotation bending test allows for high-curvature deformation with rate and temperature dependence, high data acquisition accuracy, and a small test environment to perform test programs in a time-efficient manner. Several CFRTP materials have already been characterized using this approach by several research groups for material modeling and validation purposes, including published data [14,18,22,23,24,25]. Typical bending moments for single-tape specimens at processing temperatures are in the range of 10^−4^ Nm.

Due to this low bending resistance, it must be considered that the accuracy of the measurement outcome may be sensitive to certain influencing factors. These falsify the basic assumption that the measuring moment on the rotating shaft results solely from the bending deformation of the specimen. In the PhD thesis of Sachs [20], the influences of the coefficient of friction between specimen and fixture and the clearance between the fixtures were evaluated. This was performed assuming a purely elastic beam model that deforms by bending or by shear. In conclusion, it is stated that these effects are negligible for deformation angles under 30° [20].

However, this analysis leaves a gap between the assumed ideal model conditions and the real experimental conditions. For example, the clearance during testing is fundamentally unknown due to the thermal expansion of the specimen and fixture during heating, but it can have a significant influence on the measurement [18]. Therefore, one aim of the present work is to provide further verification of the rotation bending test approach based on experimental and simulation results considering real test conditions. Based on the theoretical principles reviewed in this study, a custom-built fixture and a corresponding FE model were implemented. Following a validation procedure with a brass specimen, the focus is on the determination of the sensitivity of the test results with respect to the clearance and with respect to a pivot offset. A pivot offset may occur depending on the design of the customized fixture and when testing different specimen thicknesses.

The main objective is the characterization of the bending behavior of the PC/CF UD tape under process conditions. To the best of the authors’ knowledge, PC/CF has not been studied in this context before. Experimental testing over a wide temperature range relevant to processing allows the temperature sensitivity of the material to be determined and quantified in terms of its resistance to deformation. The data provide a basis for the optimization and analysis of the forming processes for components made of PC/CF. In addition, the data can be used to calibrate material models in non-isothermal forming simulation models.

## 2. Background of the Rotation Bending Test Method

**Figure 1:**
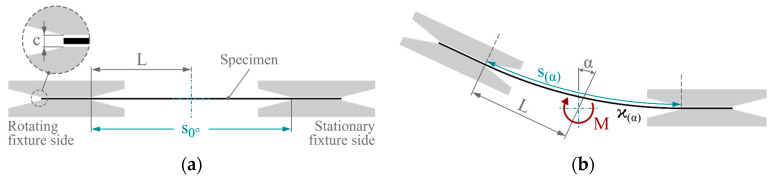
Principle of the rotation bending test: (**a**) initial and (**b**) deflected configuration.

The fundamental concept of the test method is to impose a bending deformation on a specimen using a torsional moment. For this purpose, a rectangular specimen is positioned in a two-part fixture in a gap with an adjustable clearance *c* (Figure 1a). The sample is not firmly clamped on either side. This prevents clamping effects and the superposition of spurious loads by allowing lateral movement of the specimen during bending deformation. One side of the fixture is stationary, while the second side is rotated about an axis of rotation by the angle α. During this, the specimen is bent, and the torque *M* is measured at the torsion shaft. This principle is illustrated in Figure 1b.

### 2.1. Kinematic Considerations

**Figure 2:**
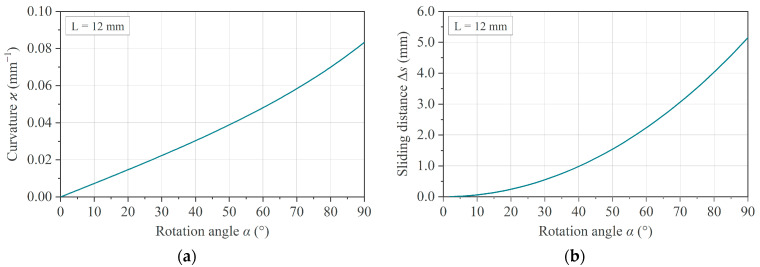
Non-linear dependence of (**a**) the curvature *ϰ* and (**b**) the lateral movement Δ*s* with increasing rotation angle, considering a constant arm length *L* = 12 mm.

Based on the stationary axis of rotation and a constant arm length *L*, the rotating side of the fixture follows a circular arc. In an idealized view, the curvature *ϰ* of a specimen under pure bending can be derived according to kinematic considerations by

(1) $$\varkappa =\frac{1}{r}=\frac{\tan\frac{\alpha }{2}}{L}\;,$$

as the reciprocal of the bending radius *r*. The effective arc length *s* of the specimen can be calculated as a function of the rotation angle α according to

(2) $$s=\frac{\alpha \;L\;\pi }{180\;\tan\frac{\alpha }{2}}\;,$$

where α is expressed in degrees, as determined during the experiments. Based on these considerations, an increasing rotation angle leads to a reduction in the effective arc length. This change in *s* during deflection with respect to the initial position follows

(3) $$\Delta s=s_{0{}^{\circ}}-\;s_{\left(\alpha \right)}=2L-\frac{\alpha \;L\;\pi }{180\;\tan\frac{\alpha }{2}}\;,$$

and can be interpreted as an indicator for the lateral movement of the specimen in the fixture. As implied by Equations (1) and (3), the dependence of *ϰ* and Δ*s* on the rotation angle α is non-linear, as shown in Figure 2.

### 2.2. Specimen Mechanics

**Figure 3:**
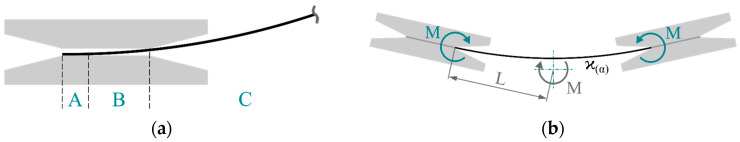
(**a**) Contact conditions between specimen and fixture and (**b**) simplified mechanical assumption.

For an overall mechanical description of the system, three sections are to be distinguished, as illustrated in Figure 3a. In section *A*, the specimen is straight and in full contact with the holder, while in section *B*, the specimen is bent up to the point of contact with the edge of the chamfer. The pure bending starts in section *C*. The analytical mechanical solution of this testing configuration requires a complex system of related equations and a numerical method with an iterative scheme to obtain a converged solution [20].

For a simplified analytical approach with respect to specimen mechanics, an idealized assumption can be made that disregards the friction effects and the space between specimen and fixture, as illustrated in Figure 3b. By applying the Euler–Bernoulli beam theory and the constant bending moment along the bending line, a relation between the bending moment *M* and the deflection angle *α* can be given by Equation (4), where *E* refers to the Young’s modulus and *I* to the area moment of inertia:

(4) $$M=\varkappa \cdot E\cdot I=\frac{\tan\frac{\alpha }{2}}{L}\cdot E\cdot I\;.$$

According to classical mechanics, the strain ($\varepsilon_{x}$) and stress ($\sigma_{x}$) value in the outer fiber of a specimen of thickness *t* along the bending direction can be expressed by Equations (5) and (6):

(5) $$\varepsilon_{x}=\varkappa \cdot \frac{t}{2}\;,$$

(6) $$\sigma_{x}=E\cdot \varepsilon_{x}=E\cdot \;\frac{\tan\frac{\alpha }{2}}{L}\cdot \frac{t}{2}=\frac{M}{I}\cdot \frac{t}{2}\;.$$

These considerations apply only for thin, homogeneous, shear-stiff, elastic test specimens (based on the Euler–Bernoulli assumptions). For inhomogeneous, shear-soft, rheology-driven samples (i.e., molten thermoplastic UD tapes), these equations do not rigorously describe the physical behavior. However, it provides a basis for the validation of the experimental set-up and a starting point for macroscopic phenomenological modeling of the bending behavior.

### 2.3. Spurious Effects

In this section, a description of the sources of spurious effects is given. Spurious effects refer to circumstances that influence the measured moment curve and lead to deviations compared to the ideal situation described above.

#### 2.3.1. Friction

**Figure 4:**
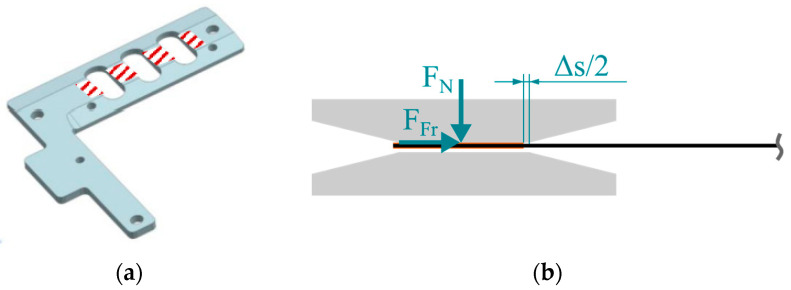
(**a**) Contact areas between specimen and fixture and (**b**) origin of friction effect.

As implied by Equation (3), the effective arc length of the specimen shortens, causing a lateral movement in the fixture with an increasing rotation angle *α*. This generates a frictional force *F*_Fr_ in the contact areas between fixture and specimen (Figure 4a), which acts as a compressive force during the deflection process, as shown schematically in Figure 4b.

The force *F*_Fr_ is non-constant, as the normal force *F*_N_ increases with the increasing bending resistance during deflection. This leads to an undesirable overestimation of the measured torque, which is dependent on the coefficient of friction (*COF*) between the specimen and fixture. As shown by Sachs [20], a *COF* = 0.5 leads to an overestimation of the torque of about 20% at a deflection angle of α = 60°, compared to the frictionless case. By applying a heat resistant polyimide (PI) tape to the contact areas of the specimen, reproducible tribological conditions can be expected. The metal/PI material pairing prevents the molten specimen from sticking and lowers the *COF* [20]. However, a valid *COF* for this material pairing, especially under high-temperature test conditions, is unknown.

#### 2.3.2. Effective Clearance

Based on the specimen thickness, the clearance *c* of the fixture must be adjusted at the beginning of a test program at ambient temperatures. During heating up to the test temperature, the specimen and the fixture are subject to thermal expansion. The effective clearance $\bar{c}$ is introduced according to Equation (7) as a temperature-dependent measure of the effective free space under test conditions.

(7) $$\bar{c}\left(T\right)={c(T)-(t}_{s}\left(T\right)+{2\cdot \;t}_{\mathrm{PI}}\left(T)\right)$$

**Figure 5:**
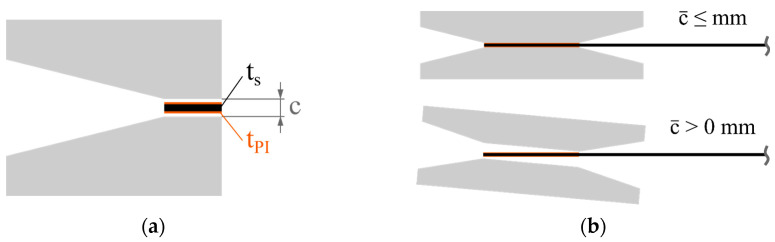
Illustration of the specimen ends positioned in the fixture: (**a**) theoretical initial situation and (**b**) effect of the effective gap $\bar{c}$ in the start phase of a test.

It refers to the difference between the clearance *c* and the specimen thickness *t*_S_, including the thickness of the polyimide tape *t*_PI_ that is applied on both sides. This is illustrated in Figure 5a.

In practice, two scenarios become possible, as shown in Figure 5b. On the one hand, if $\bar{c}$ ≤ 0, the specimen comes into full contact with the fixture and can become stuck. On the other hand, if $\bar{c}$ > 0, an idle torque in the start-up phase of the test is to be expected as long as the specimen is not in full contact with the contact surfaces of the fixture. This leads to a shift in the moment curves to higher rotation angles, as shown by Sachs with an elastic beam model [20].

#### 2.3.3. Pivot Offset

**Figure 6:**
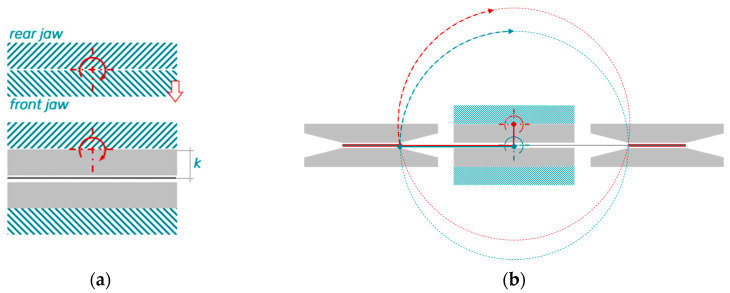
(**a**) Clamping situation leading to the pivot offset *k* and (**b**) consequence on the circular path of the rotating fixture side (circular path for *k* = 0 in blue, and for *k* > 0 in red).

To install the bending device on the rotational rheometer, the clamping unit for rectangular samples must be used. The pivot of the machine runs centrally in the parting plane of the clamping jaws in a closed position. When opening the clamping jaws, only the front jaw changes position and the rear one remains fixed. In combination with a custom-made fixture, this can result in an offset *k* between the pivot of the machine and the neutral plane of the specimen in initial position, as illustrated in Figure 6a. In this study, the offset *k* is derived as

(8) $$k=a+\frac{t_{s}}{2},$$

where a is the thickness of the fixture at the attachment area. The consequence of the eccentricity pivot is shown in Figure 6b. A deviation of the circular path of the rotating fixture and thus of the curvature compared to the theoretical considerations in Section 2.1 occurs.

The corrected curvature due to this induced offset *k* can be determined by an extension of Equation (1) according to

(9) $$\varkappa_{k}=\frac{1}{r}=\frac{\tan\frac{\alpha }{2}}{L-k\cdot \tan\frac{\alpha }{2}}\;.$$

## 3. Materials and Methods

In this section, the manufactured and implemented fixture is presented. In addition, information about the finite element (FE) model, the material to be characterized, and the experimental testing activities are given.

### 3.1. Experimental Set-Up

**Figure 7:**
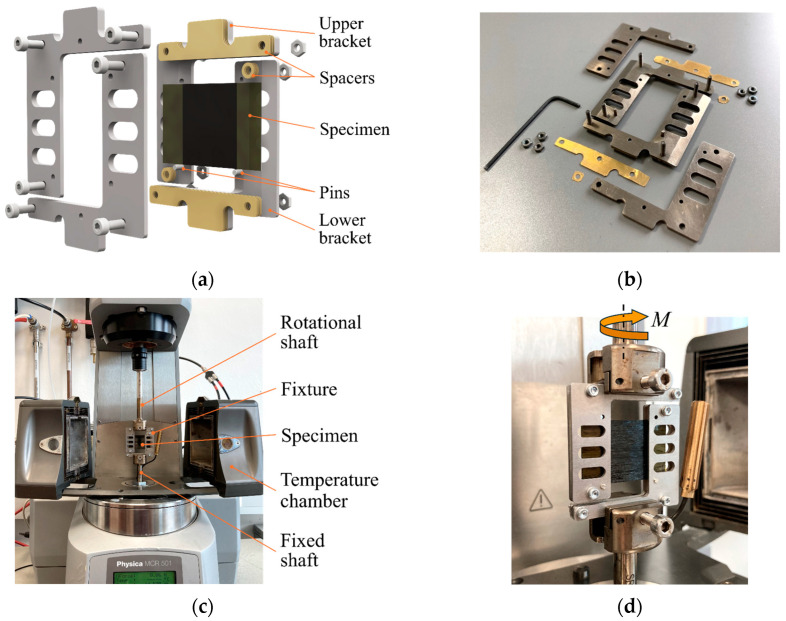
Rotation bending test: (**a**) rendering of the fixture concept; (**b**) disassembled state of the manufactured fixture showing the individual parts; (**c**) experimental set-up; and (**d**) detailed view of the installed fixture.

In the presented work, the rotational rheometer Anton Paar Physica MCR 501 (Anton Paar GmbH, Graz, Austria) was combined with the thermal chamber CTD 600 (Anton Paar GmbH, Graz, Austria). Considering the spatial limitations of the thermal chamber and the given clamping conditions, Figure 7a illustrates the concept of the fixture. The design is oriented to those used in the studies cited in Section 1 and is compatible with specimen dimensions of 22 mm × 36 mm. It consists of two parts, the upper and lower brackets, which are aligned with each other. Depending on the specimen thickness, the clearance *c* can be adjusted by metal spacers in increments of 0.1 mm. Vertical positioning of the specimen is ensured by a pin on each side. Due to the filigree characteristic of the set-up and operating temperatures of up to 400 °C, a material with a low coefficient of thermal expansion is considered beneficial for the fixture. Thus, it was made of titanium Ti-6Al-4V by laser cutting. The fixture is shown in a disassembled state in Figure 7b.

Figure 7c shows the assembled and mounted fixture in its initial state with the thermal chamber open. Figure 7d gives a detailed view of the installed fixture. The lower part of the fixture is attached to the fixed shaft, and the upper part of the fixture is attached to the rotating shaft of the rheometer.

**Figure 8:**
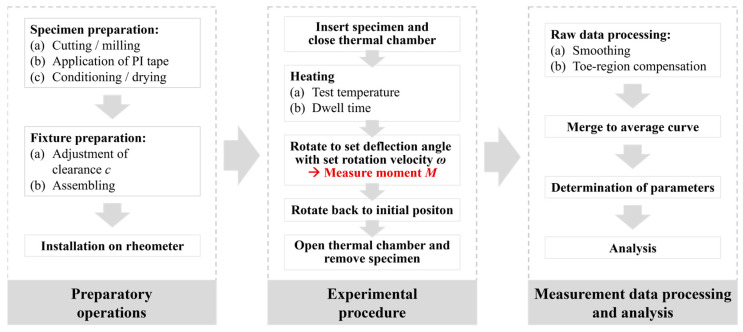
Sequence of operations for performing the rotation bending test.

Once the preparatory work has been completed, the experimental procedure can be started, as shown in Figure 8. For testing, a specimen is positioned laterally through the clearance into the fixture. After closing the thermal chamber, it is purged with nitrogen to minimize matrix material degradation, and the specimen is heated. The total heating time prior to each test is composed of the heating time of the thermal chamber to the target testing temperature plus a set dwell time to ensure a homogeneous temperature throughout the specimen. By rotating the upper shaft at a defined rotation velocity up to a defined angle of rotation, the specimen is bent. During this deflection, the applied bending moment *M* is measured. After rotating the upper bracket back to its initial position, the chamber is opened, the test specimen is removed, and a new test run can be started. The resulting moment vs. rotation angle raw data sets are finally available for processing and analysis.

### 3.2. Materials and Specimens

Two different materials were used in this study: (i) a metallic material for verification and validation of the method, and (ii) a thermoplastic composite material with a polycarbonate matrix as the material to be characterized at processing temperatures.

For the metal specimen, brass in the form of a commercially available sheet with a thickness of 0.1 mm and a specified material designation of CuZn37 was used. The material properties are summarized in Table 1 and refer to published data for the alloy used in the form of strips or sheets in accordance with DIN EN 1652 at room temperature [26,27]. Brass specimens measuring 22 mm × 36 mm were cut from the sheet.

The carbon fiber-reinforced thermoplastic UD tape material Maezio^®^ (TACF170--44GP 1003T) from Covestro (Covestro AG, Leverkusen, Germany) was investigated in this work. Table 2 gives an overview of the material characteristics provided by the manufacturer. Two specimen configurations with dimensions 22 mm × 36 mm were used. Single-ply UD tape specimens were cut from a supplied spool, and UD multi-ply specimens with the stacking sequence [0]_6_ were cut using a CNC milling machine from consolidated plates provided by the manufacturer. The ends of the specimens were cleaned with isopropanol before applying the polyimide tape.

### 3.3. Experimental Procedure

The experimental investigations are divided into three test activities. The first activity focused on validating the experimental set-up. For this purpose, a material with known mechanical properties (brass), listed in Table 1, was tested at room temperature. The experimental results were compared with the numerical results obtained from an FE model of the test set-up, using identical mechanical properties for the specimen. Following the first batch of experiments, we concluded from the good agreement of the two result sets (experimental and numerical) that the installed set-up was performing as expected, which confirmed the applicability for the determination of material properties.

The second experimental activity was aimed at investigating the influence of the effective clearance $\bar{c}$ on the experimental results. This was conducted with test batches on brass specimens at room temperature and on PC/CF specimens at 250 °C with different clearance *c* settings of the test set-up. Following the second experimental activity, we could select an appropriate setting and derive a data evaluation procedure for the following experiments.

The third experimental activity was the characterization of both single- and multi-ply PC/CF specimens at different temperatures. Based on the experimental results, the temperature-dependent bending resistance was extracted. All tests were performed at a constant deflection rate of 6°/s. The test program used is summarized in Table 3. The thickness dimensions given were determined with a micrometer screw gauge, and the set clearance dimensions were checked with a thickness gauge for each test set.

### 3.4. Simulation Model

**Figure 9:**
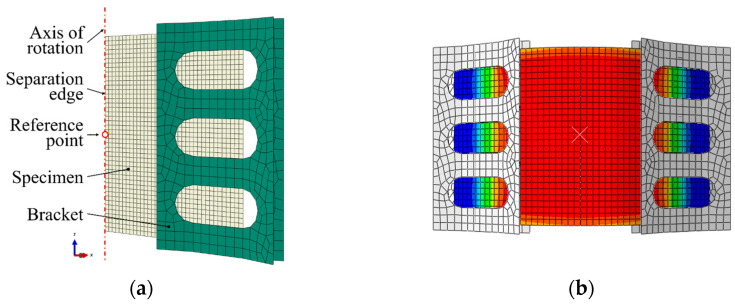
Illustration of the finite element model of the rotation bending set-up: (**a**) model set-up in ABAQUS/CAE and (**b**) representative solution.

For numerical investigations, a finite element (FE) simulation in ABAQUS/Standard (Simulia-Dassault Systems, Providence, RI, USA; Version: Abaqus/CAE 2020) according to the set-up presented in Section 3.1 was modeled. For a simple but representative model and to optimize the calculation time, it was reduced to one-half and to the relevant sections, as illustrated in Figure 9a. To ensure a kinematic motion corresponding to the experiment, the rotational degrees of freedom UR2 = UR3 = 0 and the translational degree of freedom U1 = 0 are constrained at the separation edge of the specimen. The vertical position of the specimen was ensured by the constrained translational degree of freedom U3 = 0, which was assigned to the segment of the lower edge of the specimen inside the clamp. The rotational movement of the fixture was controlled by a rotational boundary condition at a reference point (RP) located on the axis of rotation, coupled to the corresponding bracket. This same reference point was used to gather the reaction moment during the deflection. The brackets are discretized by rigid elements of type R3D4 and the specimen by S4 shell elements with a mesh size of 0.75 mm. Contact between specimen and fixture is modeled as a surface-to-surface contact interaction. An illustration of a representative solution of the FE model is given in Figure 9b.

## 4. Results and Discussion

### 4.1. Basic Validation

**Figure 10:**
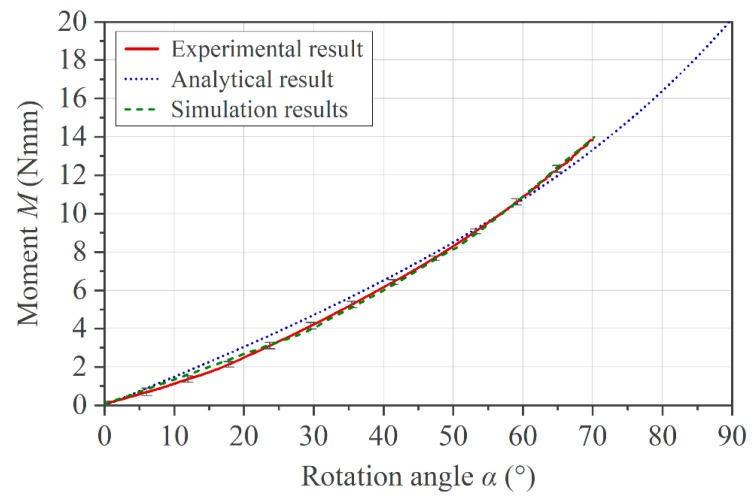
Comparison of the results between experiment, idealized analytical approach, and FE model.

A validation of the implemented set-up and the corresponding FE model was performed with a brass specimen based on the known material properties given in Table 1. Figure 10 shows the comparison between the experimental, analytical, and simulation results in terms of moment and rotation angle. All curves take into account a pivot offset *k* = 2 mm. A friction coefficient of *µ* = 0.15 was applied in the simulation, an effective gap $\bar{c}$ = 0.1 mm was set in the experimental set-up, and Equations (4) and (9) were used to obtain the analytical result. The deflection of the brass specimens was restricted to a maximum rotation angle of 70° in order to avoid a stress level above *R*_p0.2_, according to Equation (6), and thus plastic deformation of the metallic specimen.

### 4.2. Influence of the Effective Clearance $\bar{c}$

**Figure 11:**
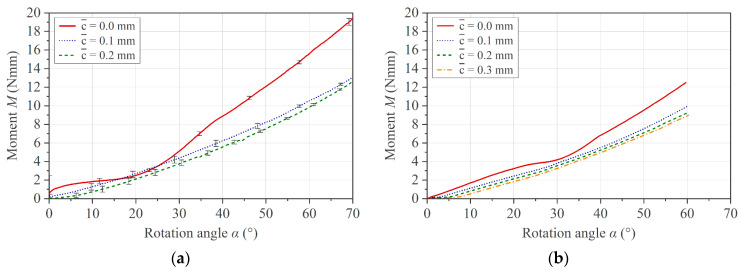
(**a**) Experimental and (**b**) simulation results on the brass specimen with respect to the influence of the effective clearance $\bar{c}$.

Based on the theoretical statements in Section 2.3.2, the influence of the effective clearance $\bar{c}$ on the measurement outcome for the brass specimens is shown in Figure 11. A too small effective gap with $\bar{c}$ ≤ 0 causes unsteady torque in the measurement, and the curve shape deviates from the other two experimental curves (Figure 11a). On the contrary, an increase in $\bar{c}$ induces a shift on the *x*-axis in the positive direction. This behavior is confirmed by the simulation, as shown in Figure 11b.

**Figure 12:**
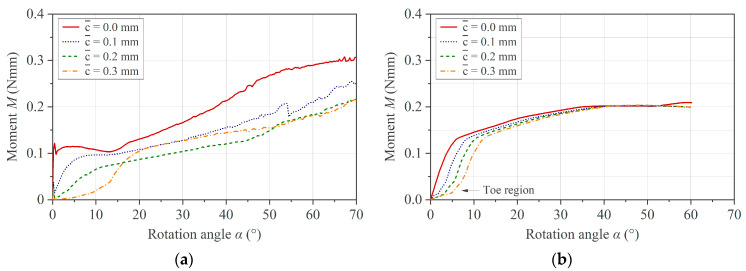
(**a**) Experimental and (**b**) simulation results with respect to the influence of $\bar{c}$ on PC/CF specimen at 250 °C.

Considering realistic test conditions for a composite specimen in the molten state at 250 °C, the effect of the clearance on the measurement result can be seen in Figure 12a. In addition to the effects described above for the metal specimen, the beginning of the momentum curve features a so-called toe region that becomes more pronounced with increasing $\bar{c}$. A toe region is known from conventional three-point bending testing and is defined according to ASTM D7264 (Standard Test Method for Flexural Properties of Polymer Matrix Composite Materials) as an artifact originating from the take-up of alignment or seating of the specimen [28]. The presence of the toe region, its dependence on $\bar{c}$, and the curve shift to higher rotation angles are again confirmed by simulation using a simple elastic–plastic material model with fitted material coefficients, as illustrated in Figure 12b.

**Figure 13:**
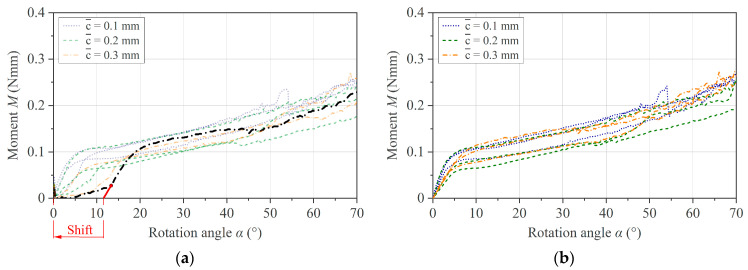
Toe region compensation applied to PC/CF experimental data at 250 °C and different $\bar{c}$: (**a**) raw experimental data set highlighting the shift of an individual measurement curve and (**b**) compensated data set.

Due to the unknown extent of thermal expansion of the specimen during melting, a sufficiently high effective clearance $\bar{c}$ must be set to avoid the artifact of an unsteady torque during the test. On the other hand, a higher $\bar{c}$ leads to a toe region and a shift in the measurement curve on the *x*-axis. The extent of both effects can vary within a test set due to variations in specimen geometry, initial settling effects of the specimen at the start of the test, or thermal expansion effects when testing at several temperatures. For this reason, we applied a correction to the individual curves before computing the mean curve and standard deviation. For the toe region compensation, a tangent is constructed from the first inflection point, which forms an intersection with the *x*-axis. The curve is then shifted to the origin. This procedure is demonstrated on raw experimental data measured with different values for the effective clearance $\bar{c}$ in Figure 13.

**Figure 14:**
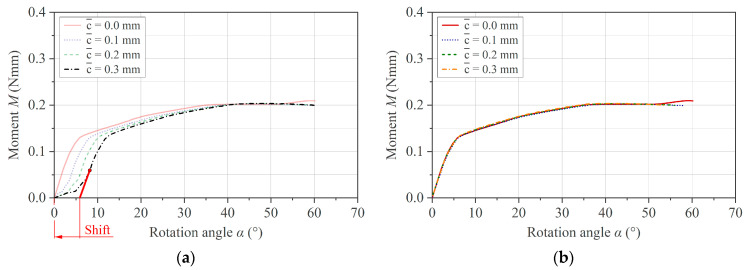
Toe region compensation applied to PC/CF simulation data with different $\bar{c}$: (**a**) simulation data set and (**b**) compensated data set.

The results of the toe region compensation applied to the data generated by the simulation are shown in Figure 14. It can be noted that the results obtained experimentally and with the simulation imply the validity of the compensation method.

### 4.3. Influence of Pivot Offset k

**Figure 15:**
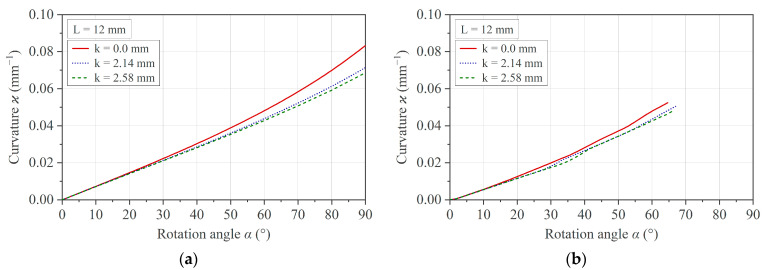
Influence of the pivot offset *k* in the curvature *ϰ*: (**a**) simplified analytical solution and (**b**) simulation result.

The pivot offset described in Section 2.3.3 is relevant for the evaluation of measurement data from specimens with different thicknesses, as *k* is larger for thicker specimens. For the measurement method used, this means that a thin specimen experiences a higher curvature *ϰ* than a thicker sample at an identical angle of rotation. Or, in other words, the curvature of a thin specimen precedes that of a thicker specimen. This effect is demonstrated in Figure 15a by plotting Equation (9) with selected *k* values and by the simulation presented in Figure 15b.

From these results, it can be deduced that the pivot offset effect can be neglected when comparing measurement curves of test specimens of different thicknesses since the measurement error at α = 30° is less than 1% and at α = 60° is less than 2%.

### 4.4. Temperature-Dependent Bending Behavior of PC/CF UD Tapes

**Figure 16:**
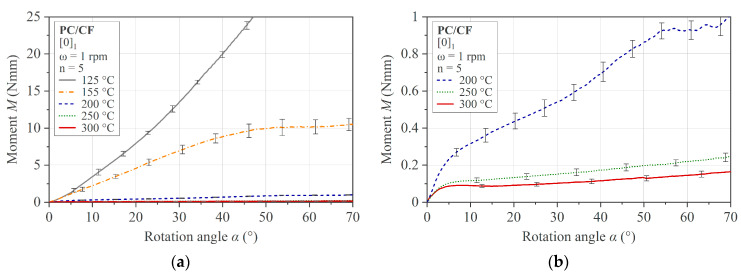
Rotation bending results for PC/CF thermoplastic UD tape single-ply specimen [0]_1_: (**a**) tested temperature range, and (**b**) domain of processing temperature range.

The average of five measurements of the single-ply specimens at five different temperatures and a constant deflection rate of 6 °/s is presented in Figure 16. The toe region compensation has been applied, and the standard deviation is given by error bars. A clear dependence of the bending behavior on the temperature is shown. As expected, the bending characteristic changes as soon as the glass transition temperature of *T*_g_ = 144 °C is exceeded. While the material shows a linear behavior in its solid state at 125 °C < *T*_g_, the curve indicates a decrease in deformation resistance and a transition to a non-linear characteristic at 155 °C > *T*_g_. A continuous non-linear decrease in bending resistance can be observed with increasing temperature, even in the processing range, as shown in Figure 16b. Another feature of the material behavior in the processing temperature range is the bilinear character of the moment–rotation angle curve, which shows two distinct regions. After a transition at a rotation angle of about 5°, a second region follows showing a consistent drop in the recorded bending resistance. From a deflection angle of about 50°, the individual measurement curves show a further drop or small irregularity. This drop is not interpreted as a material effect but as a consequence of a specimen slipping in the fixture. This reasoning is based on the disproportionate increase in lateral movement Δ*s* around this point, with reference to Section 2.1 and Figure 2. An exception to this effect is the curve at 125 °C. It can be argued that a higher frictional force in the contact areas, resulting from a higher bending resistance, prevents slippage.

**Figure 17:**
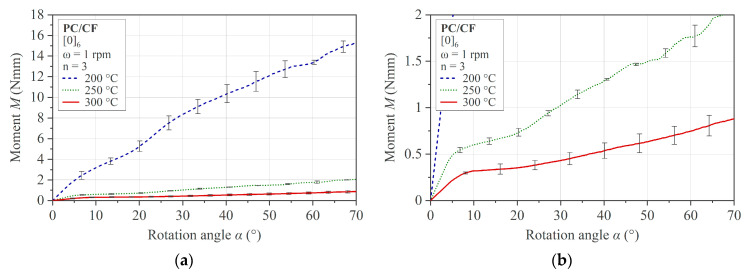
Rotation bending results for PC/CF thermoplastic UD tape multi-ply specimen [0]_6_: (**a**) tested temperature range and (**b**) enlarged domain of 200 °C and 250 °C.

**Figure 18:**
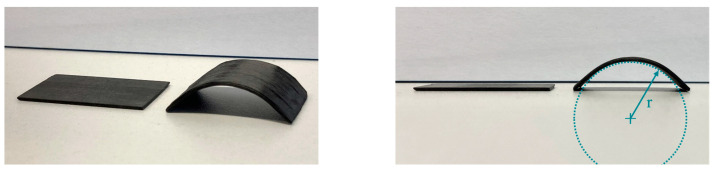
PC/CF multi-ply specimens from different camera angles, before (**left**) and after (**right**) testing.

In Figure 17, the results of the multi-ply specimens are presented. The same characteristics as in the single-ply specimens are recognizable. A comparison of a multi-ply specimen in the flat initial state and the curved state after testing is shown in Figure 18.

**Figure 19:**
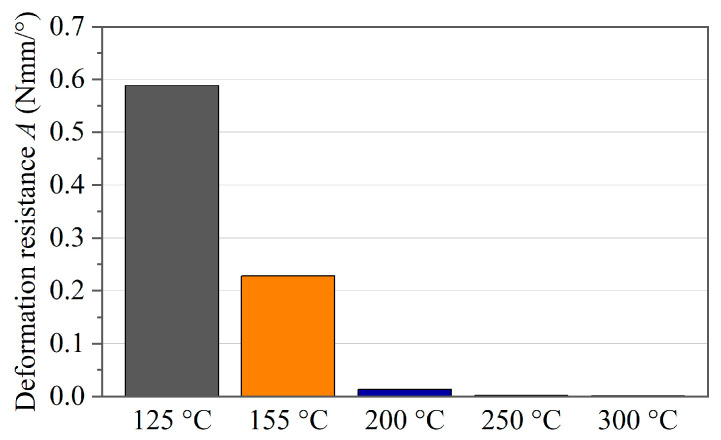
Temperature-dependent deformation resistance of a single tape.

A quantification of the deflection resistance in Nmm/° of the single-ply specimens at different test temperatures is presented in Figure 19. The measure used is the value *A*, which is determined as the slope of a linear curve fit between a 15° and 40° rotation angle. It can be stated that the bending resistance decreases by more than 97% when entering the process temperature range, compared to the material state below its glass transition temperature *T*_g_.

**Figure 20:**
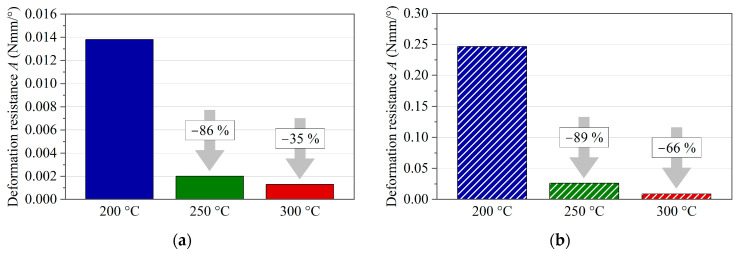
Comparison of the deflection resistances of PC/CF at different temperatures between 15° and 40° deflection angle: (**a**) single-ply specimens and (**b**) multi-ply specimens.

However, a significant temperature dependence can also be quantified in the process temperature range, as presented in Figure 20a for the single-ply specimens and in Figure 20b for the multi-ply specimens. The results show a similar temperature-dependent decrease for both sample configurations between 200 °C and 250 °C of 86% (single-ply) and 89% (multi-ply). With respect to the decrease between 250 °C and 300 °C, the single-ply specimens show a less pronounced drop of 35% than the multi-ply specimens, with 66%.

**Figure 21:**
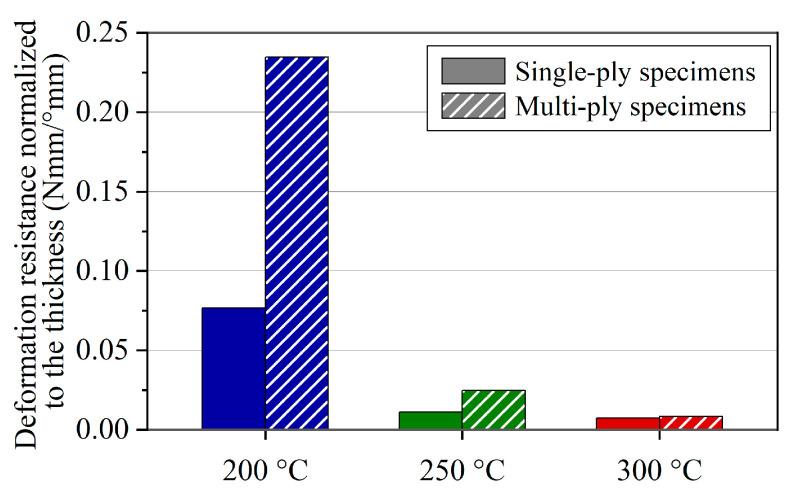
Deflection resistances normalized to the specimen thickness.

In order to evaluate the influence of the specimen thickness on the material characteristics, Figure 21 gives the deflection resistances normalized to the thickness. At lower temperatures, the multi-ply specimens show a distinctly higher material bending stiffness. On the contrary, at 300 °C, the bending resistance scales almost linearly between single and multi-ply specimens. It can be deduced from this that the shear dominance in the bending deformation increases with increasing temperature.

From a qualitative point of view, the results of the experiments carried out in the processing temperature range of PC/CF show good agreement with the published characteristic moment–deflection angle curves of CFRTPs determined by the rotation bending method [14,18,20,22,24]. The magnitude of the bending moment in the range of 0.1 to 1 Nmm for UD single-ply specimens corresponds well to the range shown in the literature [14,20,24].

The decline in the bending moment below a deflection angle of 10° is a characteristic commonly associated with molten thermoplastic polymer composites. This effect occurs when the test temperature exceeds the glass transition temperature, as the results of this study show. A similar phenomenon regarding higher deformation resistance at the beginning of the deformation process can be correlated with results obtained from ply/ply or tool/ply slippage characterization. Kapshammer et al. carried out pull-through experiments on the same PC/CF material at similar temperatures [29]. Their experimental results, obtained above the glass transition temperature, show a force peak in the initial region of the test that levels off with increasing measurement length. The correlation of these phenomena is noted here, but the thorough analysis is beyond the scope of this study, offering scope for future research.

Findings regarding the effect of the thickness of UD specimens on the bending moment and data available for thorough evaluation are scarce in the literature. Suggestive results and general assumptions that the moment scales linearly with the thickness can be found in studies [20,24]. However, the results presented here indicate that there is no linear relation for the PC/CF examined. A tendency is given for higher temperatures, but more research is necessary on this topic for a thorough understanding.

Comparing the experimental and simulation results, it can be seen that the moment–rotation angle curves are in good agreement. The FE model is able to reproduce the experimental configurations and capture the effects of model adjustments on the obtained result, such as a change in clearance. This confirms a sufficient level of modeling accuracy and points out that material properties can be extracted using this method.

## 5. Conclusions

To investigate the bending characteristics of a PC/CF UD tape material at forming temperatures, a customized bending device based on the well-known Kawabata-inspired bending method was introduced in this study. A review of the kinematics, fundamentals, and causes of spurious effects using this method was given. The influence of the effective clearance $\bar{c}$ and the pivot offset *k* on the measurement outcome was addressed using analytical, experimental, and numerical methods. While the pivot offset effect was assessed as negligible in the context of the investigated specimen thicknesses, a compensation procedure was applied for the irregular toe region start-up effect caused by a positive $\bar{c}$. The test results of the PC/CF UD tape material show a distinct temperature dependence of the material’s deflection resistance within the proposed processing window. A drop of about 88% between 200 °C and 250 °C and a further decrease of averaged 50% between 250 °C and 300 °C were observed. With regards to practical thermoforming processes, it can be deduced that material deformation above 250 °C is beneficial due to the markedly lower bending resistance, especially for complex geometries. The presented data can be used for constitutive modeling of the PC/CF material at forming conditions. Rate-dependent testing should be investigated for this thermoplastic composite material in the next step to capture the influence of the deformation rate on the deformation resistance.

## Figures and Tables

**Table 1:** Material properties of brass specimen from data sheet [26].

Property	Unit	Value
Material type	-	Brass (CuZn37, CW508L)
Density	g/cm^3^	8.44
Modulus	GPa	110
*R*_p0.2_	MPa	370
Poisson’s ratio	-	0.37
Sheet thickness	mm	0.1

**Table 2:** Material properties of the investigated PC/CF tape material.

Property	Unit	Value
Polymer matrix type	-	Polycarbonate (Makrolon^®^) ^1^
Fiber type	-	Carbon
Fiber volume content	%	44
Density	g/cm^3^	1.5
Glass transition temperature	°C	144
Nominal tape thickness	mm	0.175
Tape width	mm	120
Processing temperature	°C	180–300

^1^ Exact material grade not specified.

**Table 3:** Overview of the test program configurations.

		Pre-Tests		Characterization	
Specimen Type		Brass	PC/CF Single-Ply	PC/CF Single-Ply	PC/CF Multi-Ply
Lay-up	-	-	[0]_1_	[0]_1_	[0]_6_
Specimen thickness	mm	0.10	0.18	0.18	1.05
PI-covered thickness	mm	0.19	0.27	0.27	1.15
Set clearance *c*	mm	0.2, 0.3, 0.4	0.3, 0.4, 0.5, 0.6	0.4	1.50
Effective clearance $\bar{c}$	mm	0.0, 0.1, 0.2	0.03, 0.13, 0.23, 0.33	0.13	0.35
Soaking time	min	-	3	3	8
Repetitions	-	3	5	5	3
Temperature	°C	23	250	125, 155, 200, 300	200, 250, 300
Deflection rate	1/s	6°	6°	6°	6°

## Data Availability

Regulated by the compliance with our cooperative research framework, not all data can be made publicly available. However, for research purposes, data presented in this study will be available upon request from the corresponding author.
